# The Global Advance of Genome-Edited Plants to the Market: The Key Role of Chile in Its Development

**DOI:** 10.3390/plants13243597

**Published:** 2024-12-23

**Authors:** Miguel A. Sánchez

**Affiliations:** ChileBio CropLife, Antonio Bellet 77, Of 607, Providencia, Santiago 7500025, Chile; masanchez@chilebio.cl; Tel.: +56-2-2235-4001

**Keywords:** genome editing, biotechnology, plants, new breeding techniques, regulatory frameworks, Chile

## Abstract

The global advancement of genome-edited plants toward commercialization has been significantly shaped by the functionality and flexibility of some regulatory frameworks governing plant genome editing. These frameworks vary widely across countries, reflecting diverse approaches to assessing and managing the risks and benefits of genome-editing technologies. While some nations have adopted product-based frameworks that focus on the characteristics of the final plant rather than the technique used, others rely on more restrictive process-based regulations. This variability influences the pace of innovation, the types of products able to enter the market, and their global trade potential. Chile stands out as a leader in this landscape, having implemented a science-driven and flexible regulatory framework. Its system promotes innovation by facilitating genome-edited plant research and development, field testing, and local commercialization. This regulatory adaptability positions Chile as a critical player in supporting the global integration of genome-editing technologies into agriculture, fostering advancements that address food security, sustainability, and climate resilience.

## 1. Introduction

New Breeding Techniques (NBTs) such as genome editing are transforming modern agriculture by enabling precise modifications to plant genomes that can accelerate breeding [1,2], enhance crop resilience [3,4], and improve nutritional quality [5,6]. Technologies like CRISPR-Cas9, TALENs, and other gene-editing tools fall under NBTs scope and offer promising alternatives to conventional breeding and traditional genetic modification, often involving transgenic approaches [7]. These advancements are particularly valuable in addressing the urgent challenges of climate change, environmental sustainability, and food security, which have become critical concerns in agriculture worldwide [8].

Since 2015, several countries have updated their regulatory frameworks for genetically modified organisms (GMOs) or applied other regulatory approaches to harness the potential and benefits of these technologies and adopt them in the agricultural/food value chain. However, countries vary their approaches to regulating these technologies, influencing their frameworks’ functionality, adaptability, and flexibility [9,10].

In 2017, Chile became the second country globally, following Argentina, to establish a regulatory framework for plant products developed through NBTs. Chile’s regulatory stance on NBTs is influenced by its commitment to international trade, alignment with global biosafety standards, and the need to maintain competitiveness in an increasingly biotechnology-driven agricultural sector [11].

In Chile, a country known for its diverse agricultural production and major role in global markets for fruits, wine, and seeds, along with research activities for global plant breeding programs, NBTs hold significant potential for improving crop productivity and resilience. However, the success of these technologies for the country is tightly coupled with the regulatory frameworks that govern them, not only in Chile but in different regions of the world. The regulatory landscape for NBTs in Chile, as in many countries, is evolving to address the specificities of genome editing and other advanced biotechnologies, which differ fundamentally from traditional GMOs [9]. Given that genome-edited crops can often be developed without introducing foreign DNA, making them untraceable and undistinguished from conventional crops, they present a regulatory challenge, with questions about how these products should be assessed, classified, and commercialized.

This article aims to provide an in-depth analysis of the regulatory landscape for NBTs in Chile, examining the criteria used for NBT classification, the evaluation processes, what products have been reviewed by regulators, and the considerations for emerging products. Furthermore, it tries to provide an analysis of the pace of adoption of NBT-derived products in countries with functional regulatory frameworks. By focusing on Chile, the article offers insights into the challenges and opportunities faced by countries that aim to promote agricultural innovation while navigating complex regulatory requirements. Understanding the regulatory environment for NBTs is essential not only for local developers but also for international stakeholders who are keen to invest in or collaborate with Chile’s agricultural biotechnology sector.

## 2. The Regulatory Framework in Chile

The Agricultural and Livestock Service (SAG), a regulatory agency depending on the Ministry of Agriculture, oversees the regulation of agricultural biotechnology. Although Chile has not ratified the Cartagena Protocol (CP), it uses the CP GMO definition for its regulatory approach for NBTs, considering a GMO as any living organism with a novel combination of genetic material derived through modern biotechnology [12]. Given that NBTs inherently involve modern biotechnology (i.e., genetic engineering) and that the CP does not specify what constitutes a “novel combination of genetic material”, Chile clarified this concept to give certainty to breeders and public and private developers. It was defined as a stable insertion of one or more genes or DNA sequences that encode proteins, RNA interference, double-stranded RNA, signal peptides, or regulatory sequences introduced permanently into the plant genome. Unlike traditional GMOs, where foreign DNA is inserted into the plant genome, NBT-derived products can bear genetic changes without necessarily having exogenous DNA in the final product. Thus, these products do not fit the GMO definition, and according to Chile’s approach, they are considered conventional.

The regulatory approach is not an authorization process but is a determination, on a case-by-case basis, whether a plant product is a GMO or not. It determines just whether a plant product developed through genetic engineering contains a novel combination of genetic material (foreign DNA). The approach is open to any biotech tool and does not represent a risk assessment. The application form consists of two sections, including (a) applicant information and (b) technical information (taxonomy, cultivar/lines, phenotype, biotech technique used, determination of absence of foreign DNA, and indication if the propagation material has been authorized by the official agency of any country). The response time is 20 working days [13]. The regulations have been praised for their simplicity, speed, and predictability.

## 3. Key Insights About the Metrics of Chile’s NBT Regulatory Process

Since the regulatory framework was introduced in 2017, 53 submissions have been received. It is worth noting that before the regulatory approach was implemented, another four consultations were requested. All the information was obtained through a request made to SAG under the Chilean Transparency Law. Thus, as of November 2024, out of a total of 57 submissions, 52 consultations have been deemed non-GMO due to the absence of foreign genetic material, and 5 consultations were concluded to be GMOs. This fact highlights the framework’s efficiency in distinguishing between GMO and non-GMO genome-editing applications, and the significant number and pace of consultations. This high proportion of non-GMO classifications underscores the adaptability of the framework to facilitate innovation in plant breeding by streamlining the regulatory process for crops that do not involve foreign DNA. The five cases deemed to be GMOs provide valuable insight into the nuanced application of the regulations. One submission was classified as a GMO because, despite the plant product being developed using cisgenesis, where a gene from a closely related species was introduced, a promoter from a non-sexually compatible species was used. There was another case where the genetic material of the CRISPR/Cas system was still inserted in the plant genome, making it considered a GMO. The remaining three situations involved genome-edited GMOs. In these cases, the genome-editing process did not add foreign DNA; however, the genome-edited plants had originally been modified using genetic engineering methods and bore a transgene, which meant the final products were still considered GMOs. These examples emphasize the Chilean framework’s ability to evaluate genome-edited products on a case-by-case basis, ensuring that regulatory decisions are based on the specific characteristics of each application. This balance of supporting innovation while maintaining a robust assessment framework enhances confidence among developers and stakeholders in the system’s fairness and scientific rigor.

**Figure 1:**
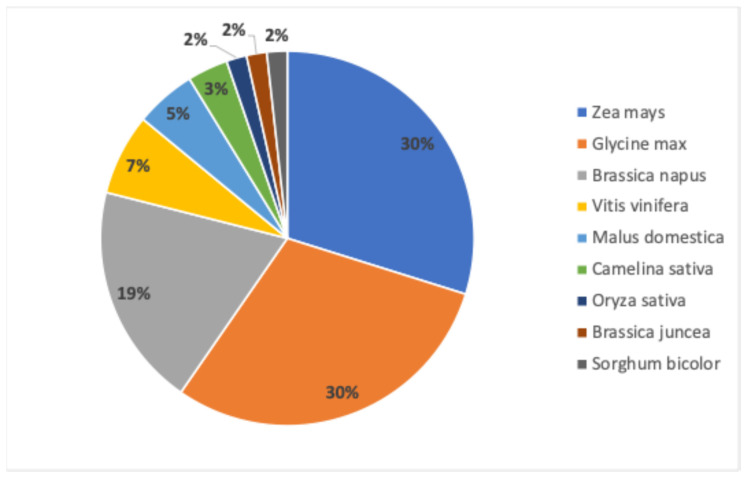
Relative percentage of crop species included in the 57 submissions in the NBTs Chilean regulatory framework submissions.

**Figure 2:**
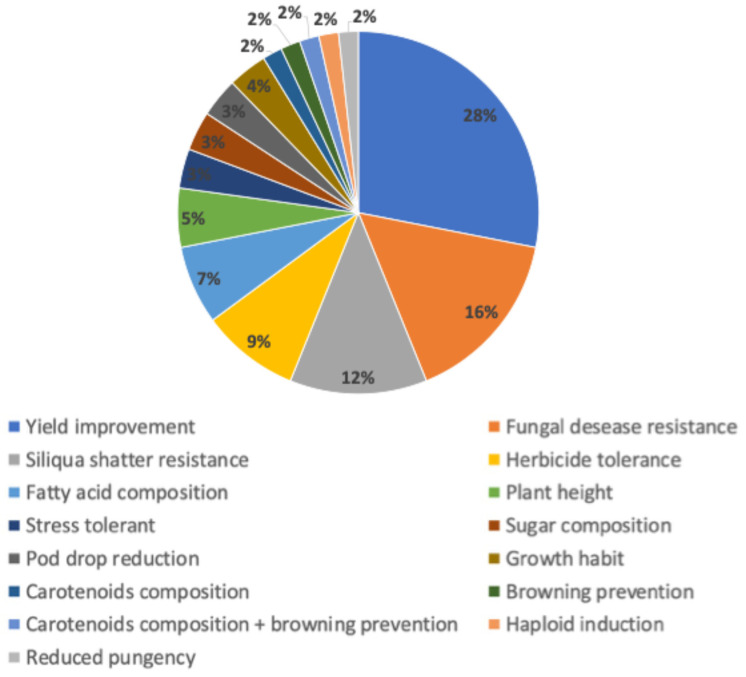
Relative percentage of phenotypic traits targeted included in the 57 submissions in the NBTs Chilean regulatory framework.

Nine different crop species have been evaluated from the 57 submissions that navigated the Chilean regulatory framework, the most common being maize and soybean (Figure 1). In addition, submissions have predominantly focused on traits that enhance yield. Fungal disease resistance and pod shatter resistance are other traits of interest. Some applications also target improved nutritional profiles or other consumer-oriented characteristics (Figure 2).

**Figure 3:**
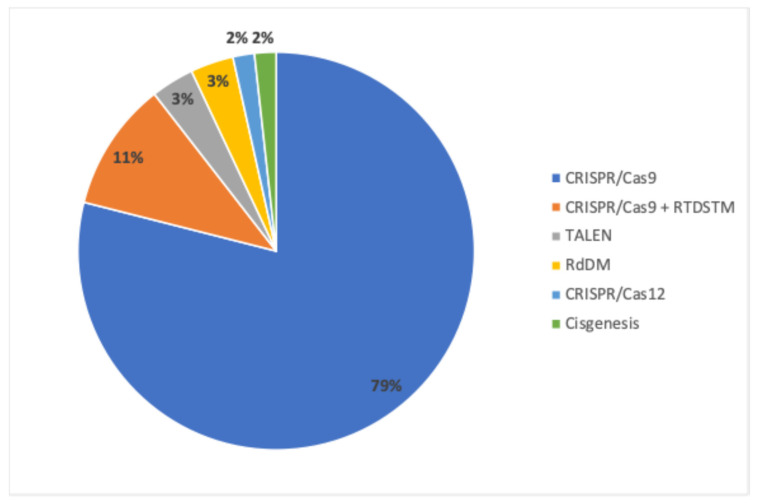
Relative percentage of NBTs used to develop new cultivars/lines included in the 57 NBT Chilean regulatory framework submissions.

Among the applications, CRISPR-Cas9 remains the most popular method, perhaps due to its precision and versatility. Further, Cas9 may be the most adaptable Cas currently available [14,15]. Other techniques, such as Rapid Trait Development System (RTDSTM), TALENs, and RNA-directed DNA methylation (RdDM), have also been employed, reflecting global trends in genome editing preferences (Figure 3). RTDSTM is a mutagenesis technology that uses the natural or inherent mismatch-repair system to effect a genetic change [16].

**Figure 4:**
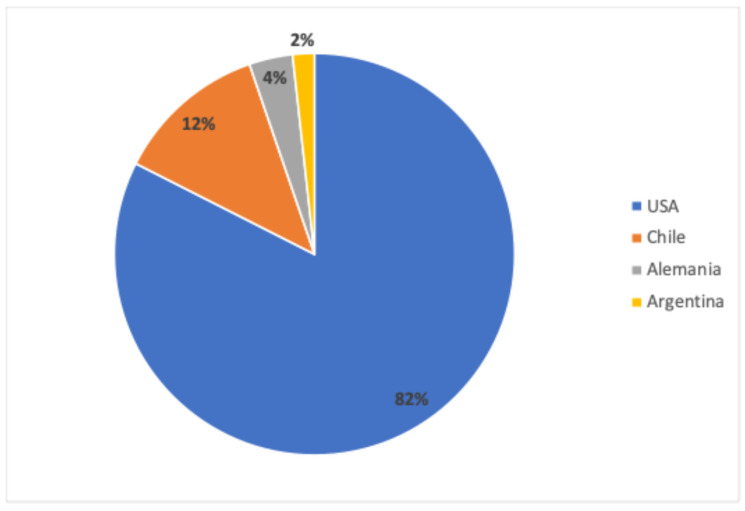
Relative percentage of the country of origin of the 57 submissions that have navigated the NBTs Chilean regulatory framework.

Chile has attracted submissions of NBT-derived plant products from both local and foreign developers. Applicants come from a few countries, where 7 out of the 57 applications have come from local institutions, while 50 have come from foreign entities, reflecting a broad international interest in utilizing Chile’s regulatory pathway (Figure 4). This international interest reflects Chile’s favorable regulatory environment for innovation in plant breeding [11].

Typically, submissions in Chile involve multiple lines/cultivars per application, with some using multiplexing approaches to achieve a variety of edits in a single genome. According to the Chilean NBTs regulatory framework, for multiple lines derived from the same editing process and for multiple edits in the same gene or different genes, only a single form must be submitted that considers the construct(s) used, and all the lines presented in the form must be associated with the same phenotype [13].

From the 57 applications already submitted to Chile’s NBT regulatory system, it is worth noting that these included not 57 but 1103 lines. Only 21 applications (37%) include only one line/cultivar to be evaluated by the SAG. Interestingly, 16 applications contain more than 10 lines, 6 have more than 50, and 4 have more than 100. The application that included the largest number of lines was one with 270.

On the other hand, submissions indicate a trend toward multiplexing, where several genes are edited simultaneously to create complex phenotypes or one single gene is edited in different regions. This capability is increasingly important for developing multifaceted traits, such as climate resilience and increased yield [17,18]. In the case of Chile’s NBT regulatory framework, 51% of the 57 submissions have included a multiplexing strategy.

Submitting multiple lines obtained from a single genome-editing process under a single application form carries significant advantages for both developers and regulators. This approach streamlines the evaluation process, reducing administrative burdens and accelerating the time to market for genome-edited crops. From the developer’s perspective, it allows for the inclusion of variations that arise from the same editing event, such as lines with different degrees of trait expression, without needing separate applications. For regulators, evaluating multiple lines together provides a comprehensive view of the editing outcomes, enabling a more efficient assessment of compliance with regulatory standards. This consolidated approach is particularly beneficial for techniques like multiplex genome editing, where several genetic changes are introduced simultaneously, leading to the generation of numerous lines with potentially valuable traits.

Chile’s regulatory approach has drawn significant interest from both local and international developers, positioning the country as a leader in agricultural biotechnology within Latin America. By allowing case-by-case determinations, Chile’s framework remains adaptable to new genome-editing techniques, providing a robust system that fosters innovation.

## 4. The Pace of Consultations of NBT-Derived Plant Products in Other Countries

The adoption of plant NBTs is advancing globally, driven by their potential to address issues of food security, sustainability, and climate resilience. However, the regulatory frameworks governing genome editing vary significantly across regions, influencing the speed and extent of technology adoption (Table 1) [9].

For instance, the United States has taken a product-based approach, focusing on the characteristics of the final product rather than the process used to create it. The U.S. Department of Agriculture (USDA) exempts from GMO regulation those modifications that could otherwise be achieved through conventional breeding: (i) change resulting from cellular repair of a targeted DNA break in the absence of an externally provided repair template; (ii) the genetic modification is a targeted single base pair substitution; or (iii) the genetic modification introduces a gene known to occur in the plant’s gene pool or makes changes in a targeted sequence to correspond to a known allele of such a gene or to a known structural variation present in the gene pool [19]. This flexibility has fostered a favorable environment for innovation, with a significant number of genome-edited plants that have been exempted from GMO regulation. To help developers put their products on the market, the USDA has implemented a procedure for those entities to voluntarily request a confirmation letter that a plant is exempt from the regulation. For developers not seeking confirmation letters, no submission of information to the USDA is required. As of November 2024, 99 confirmation requests have been submitted to the Animal and Plant Health Inspection Service (APHIS) since 2021 [28]. The U.S. regulatory framework has been useful in promoting confidence among stakeholders and encouraging both local and international developers to conduct research and commercialization within its borders. Four genome-edited plants have been introduced to the U.S. market: (i) SU Canola™, a sulfonylurea-tolerant rapeseed developed using oligonucleotide-directed mutagenesis [29]; (ii) Calyno™ oil-producing soybean, created with TALEN technology, featuring a high oleic acid content, reduced saturated fat, and no trans fats [30]; (iii) Conscious™ greens, mustard greens edited with CRISPR to reduce pungency [31]; and (iv) GreenVenus™ romaine lettuce, a non-browning variety that stays fresh and crisp for up to two weeks longer than conventional types [32].

On the other hand, Canada has always implemented a novelty-based regulatory framework for plants with novel traits, and recent regulatory updates have provided critical clarifications, including how products developed with genome editing fit into the framework. The Canadian regulatory system is based on the product, not the process used in its development. It allows product developers and plant breeders to self-determine the novelty of their traits against specific triggers depending on the end use. This approach covers all novel products regardless of the process used in their development (traditional breeding, transgenesis, or genome editing). The presence of foreign DNA in the final plant product is a key aspect for determining the novelty for food, feed, and environmental release. However, a few other end-point-specific risk-based criteria are considered. The Canadian Food Inspection Agency (CFIA) and Health Canada evaluate novel traits for potential risks to human health, safety, and the environment. If a genome-edited crop does not exhibit a novel trait, it is exempt from further regulatory scrutiny, streamlining the approval process. However, Canada’s approach considers that plants with a new commercially viable herbicide tolerance trait (but no inclusion of foreign DNA) always require authorization [20]. Health Canada has introduced a voluntary transparency initiative that allows developers of gene-edited plants intended for food use, even if they were not considered novel foods, to disclose their products, promoting greater openness and consumer confidence. As of October 2024, there is a list of non-novel products of plant breeding intended for food use in Canada containing 14 notifications [33].

Based on the National Technical Biosafety Commission (CTNBio) Normative Resolution No. 16, Brazil has established a case-by-case consultation process to determine whether a product obtained by NBTs should or should not be classified as GMO. The criteria to exclude products from GMO regulations include the following: (i) absence of recombinant DNA/RNA; (ii) presence of genetic sequences that could be obtained by conventional breeding (crossing); (iii) presence of mutations that could be obtained by mutagenesis; and (iv) presence of mutations that could occur spontaneously in nature. As of November 2024, and since 2018, CTNBio has concluded that 16 consultations about NBT-derived plant products do not represent GMOs [21].

In 2018, Colombia adopted a regulation that distinguishes genome-edited plants from GMOs based on the presence or absence of foreign DNA. As of November 2024, eight consultations about NBT-derived plant products have been submitted to the regulatory system [34].

Argentina was the first country globally to address the topic by regulating products derived from NBTs in 2015, and its regulators have contributed actively to technical and regulatory advancements for NBTs in South America, Africa, and Asia. Under the scope of the National Advisory Commission for Agricultural Biotechnology (CONABIA), a product-based approach has been established to analyze whether a new combination of genetic material is generated in plants, animals, and microorganisms. Consultations to be analyzed by CONABIA can include fully developed products or early-stage developments. In this last situation, the applicant must later submit a second form when the product is finished to verify whether the genetic changes introduced coincide with those described in the first consultation. CONABIA has 80 working days to give an official response, concluding if the product is GMO or conventional [23]. Since 2015, CONABIA has received 92 consultations related to fully developed NBT-derived plants and 32 consultations for plants in early-stage development (F. Simeone, CONABIA, personal communication, 20 November 2024).

In Kenya, as of October 2024, the National Biosafety Authority (NBA) has approved three plant genome-edited products for research purposes [35]. In 2022, NBA regulated genome-editing techniques establishing guidelines to provide clarity on which genome-edited organisms and/or derived products should be regulated under the Biosafety Act and which products would be exempted and managed as conventional varieties or breeds In a decision made on a case-by-case basis, the approach exempts the following cases: (i) all modifications made by inserting genes from sexually compatible species and where regulatory elements (promoters and terminators) are also from the same species; (ii) all deletions/knockouts, provided that there is no insertion of foreign genetic material in the end-product; (iii) processed products whose inserted foreign genetic material cannot be detected; and (iv) conventional breeding methods, mutagenesis, polyploidy, and haploidy. Interestingly, no registration of a local entity with the regulatory agency is necessary to initiate a submission to the regulatory body [24].

In 2019, Japan’s Ministry of Environment and Ministry of Health, Labour and Welfare (MHLW) established guidelines that classify genome-edited plants differently from GMOs, provided they do not contain foreign DNA. Genome-edited plants with simple edits, such as deletions, insertions, or single nucleotide changes that could occur naturally, are exempt from GMO regulations and subject only to notification procedures. Developers must inform authorities about the product and its intended use, but these plants do not require lengthy risk assessments or labeling as GMOs. However, information about changes in traits and possible impacts on biodiversity is required. Genetic modifications that involve the insertion of extracellularly produced nucleic acid (template) are still considered GMOs, even though the mutation produced is equivalent to those that occur naturally. As of November 2024, two genome-edited plants have been notified to the Ministry of the Environment and the MHLW. These are tomatoes with high gamma-aminobutyric acid (GABA) accumulation (two bred lines) and waxy corn [25]. Currently, the high-GABA tomato is marketed in Japan. It was developed using CRISPR/Cas9 technology and contains up to five times higher levels of GABA, which is a naturally occurring non-protein amino acid that functions as a neurotransmitter in the nervous systems of humans and other mammals. Some studies suggest that GABA may help lower blood pressure, potentially offering cardiovascular benefits [36].

The Memorandum Circular No. 08 Series of 2022 sets out the rules and procedures for the evaluation of novel plant breeding technologies of the Philippine Department of Agriculture. Biotech final products may be deemed non-GMO if they do not contain a novel combination of genetic material. Developers must request a confirmation from the Bureau of Plant Industry (BPI) to receive a certificate letter that the BPI does not consider an NBT-derived plant product to be regulated according to the Circular establishing regulatory policies for GMOs (JDC-1). The certificate shall not excuse the developer from complying with other relevant regulations, such as those involving quarantine, pest risk analysis, varietal registration, and crop-specific standards/programs [26]. As of November 2024, three certificates have been granted, two for different varieties of reduced-browning bananas and one for gene-edited high-GABA Sicilian Rouge Tomato [37].

China’s regulatory framework for genome-edited plants reflects an extremely cautious approach to agricultural biotechnology, contrary to the trend of regulatory approaches in the rest of the world. In 2022, the Ministry of Agriculture and Rural Affairs (MARA) introduced guidelines that distinguish genome-edited crops from GMOs if they do not involve the introduction of foreign DNA [27]. However, under this framework, genome-edited products fall within the scope of GMO regulations and are regulated as GMOs. NBT-derived plant products must get a safety certificate after field testing, safety assessments, and final approval from MARA. Based on a case-by-case analysis, genome-edited crops without foreign DNA are classified into four categories regarding the risk profile of the trait of interest. Instead of the almost 10 years required for GMO safety certificates, NBT-derived plant products can obtain a safety certificate in 1–2 years [27]. Different information is required based on whether the NBT-derived plant may directly change the relationship between species. If no impact is expected, it is necessary to provide (i) target traits and evaluation of functional efficiency and (ii) survival competitiveness. If an impact is expected, then it is necessary to add (iii) impacts on the ecosystem community structure and evolution of pest status. (iv) Gene-edited plants resistant to diseases and insects should also provide indoor bioassays for non-target organisms that may be affected. Finally, (v) herbicide-tolerant gene-edited plants should also provide tolerance to at least three other commonly used (non-target) herbicides. At the same time, if a gene-edited plant may have changed its key components, it is necessary to provide (i) an analysis of key components; (ii) if gene editing leads to a significant increase in the expression of a certain protein or the generation of a new protein, a corresponding safety evaluation should also be performed on the protein; (iii) if the above data indicate that the target trait may increase food safety risks, a 90-day feeding test on rats should also be provided. Furthermore, (iv) an assessment may be required of the impact of the maximum possible intake level on the dietary pattern of the population [38]. As of November 2024, MARA has issued five safety certificates for NBT-derived plant products, including soybean (3), corn (1), and wheat (1) [39,40,41].

## 5. Challenges in the Implementation of NBT Regulations

The complex international landscape surrounding genome-editing technologies and the diversity of products now emerging from NBT applications shape pre- and post-market challenges. Pre-market challenges in the regulation of NBTs present significant hurdles for developers, often delaying the path to commercialization. A notable issue is the lack of consultations for early-stage developments, where developers face uncertainty regarding regulatory expectations for their products. This gap can lead to inefficiencies in data generation, resulting in time and resource waste. Chile and other countries may strengthen their regulatory approaches to mirror Argentina, which stands out as an example of a country with a regulatory system that allows the consultation of early-stage products. Additionally, inconsistent data requirements across different regulatory frameworks create further complications, as developers must navigate varying standards for safety and efficacy assessments. This challenge is particularly pronounced for genome-edited products with highly similar traits, which may require multiple regulatory status determinations despite sharing the same underlying modifications. These repeated evaluations can increase costs and prolong approval timelines, discouraging innovation. Addressing these challenges through harmonized regulatory guidance, streamlined processes, and opportunities for early engagement with regulatory agencies could significantly reduce barriers and foster a more efficient path to market for NBT-derived products.

Because of NBT-derived products are indistinguishable from conventional varieties, the demand for post-market requirements such as labeling, traceability, segregation, and monitoring are nonsensical.

As presented above, some regulatory frameworks for genome-edited crops differ significantly across countries, which may cause market access issues for developers. For instance, the divergence of Chile’s robust and efficient NBT regulations from other frameworks, especially in Asia, limits the export potential of genome-edited crops, creating uncertainty for local and foreign developers.

Most current regulatory models are primarily focused on determining the presence/absence of foreign DNA to classify products as either GMO or non-GMO. While this approach has proven effective for many genome-edited crops to date, the rapidly evolving techniques and the emergence of more complex genetic modifications, such as translocations, inversions, and changes in gene copy number, among others, present new challenges. Products involving these higher-order edits (beyond simple deletions or insertions) are already on the horizon, aiming to address more complex agronomic traits, such as those controlled by multiple genes. The current regulatory structure does not account explicitly for these products, which could lead to delays or regulatory hurdles as developers seek approval. Additionally, the lack of international harmonization in addressing these novel modifications may lead to discrepancies between countries, complicating global trade and market access for these products. To remain adaptive and scientifically robust, Chile’s regulatory system, along with others worldwide, may need to consider updates that account for these complex modifications while fostering alignment with international standards.

On the other hand, public perception and market acceptance of NBT-derived products play a critical role in the development and adoption of genome-edited crops, often proving to be as influential as regulatory frameworks. Even in countries like Chile, with favorable and science-based regulations, public skepticism or misunderstanding about genome editing can impede progress. Concerns about the safety of NBTs, confusion with traditional GMOs, and a lack of awareness about the benefits of these technologies can lead to resistance from consumers, retailers, and policymakers. This, in turn, affects market demand and the willingness of agricultural producers to invest in these innovations. Furthermore, export-driven agricultural economies like Chile’s are particularly vulnerable to international market preferences, which may impose stricter standards or reject genome-edited products regardless of their regulatory status. Addressing these challenges requires transparent communication, public engagement, and education campaigns that emphasize the safety, sustainability, and economic benefits of NBT-derived crops. Without such efforts, the full potential of favorable regulatory frameworks could remain unrealized, hindering the development and commercialization of innovative agricultural solutions.

## 6. Conclusions

Chile’s product-based regulatory framework positions it among the more innovation-friendly regulatory regimes, attracting international developers with its clear, streamlined processes for non-GMO classified products. This system, based on the presence or absence of foreign DNA—a widely accepted global criterion and the primary standard for distinguishing genome-edited plants from GMOs—provides functionality, flexibility, and predictability.

Chile’s regulatory model excels in accommodating innovations such as multiplexing and the development of multiple lines per editing process, encouraging plant breeders to conduct field tests and locally multiply seeds in the counter-season. However, challenges persist in achieving international regulatory alignment and addressing emerging complexities in genome-editing techniques to facilitate global trade and the import/export of NBT-derived plant products.

Chile must proactively adapt its framework to maintain its leadership, balancing innovation with safety and market demands. Strengthening collaboration with global regulatory bodies and scientists will help align practices with international standards while retaining flexibility for novel NBT products. Transparency and public engagement regarding the benefits and safety of genome editing will be vital to fostering acceptance and maximizing the impact of NBTs for sustainable agriculture.

## Figures and Tables

**Table 1:** Summary of regulatory frameworks and progress in different countries regarding NBT-derived plant products.

Country	Framework Type	Regulatory Triggers	Number of Consultations/ Notifications Considered Non-GMO	Key Features	Reference
Chile	Product-based	Novel combination of genetic material	52	Case-by-case basis, allows multiplexing and multiple lines per application.	[13]
USA	Product-based	Traits not achievable via conventional breeding	99	Voluntary confirmation letters, streamlined exemptions for conventional-like edits.	[19]
Canada	Novelty-based	Novel traits, including herbicide tolerance	14	Self-determination of novelty, voluntary disclosure for transparency.	[20]
Brazil	Product-based	Presence of recombinant DNA/RNA	16	Case-by-case consultation.	[21]
Colombia	Product-based	Novel combination of genetic material	8	Case-by-case consultation.	[22]
Argentina	Product-based	Novel combination of genetic material	92 (fully developed) + 32 (early stage)	Case-by-case consultation. First country to regulate NBTs.	[23]
Kenya	Product-based	Novel combination of genetic material	3	Case-by-case consultation.	[24]
Japan	Product-based	Novel combination of genetic material	2	Case-by-case consultation. Regulations formulated by the Ministry of the Environment and the Ministry of Health, Labour and Welfare.	[25]
Philippines	Product-based	Novel combination of genetic material	3	Case-by-case consultation.	[26]
China	GMO-based	All products are classified on the risk profile of the target trait	5	Cautious approach, complex requirements for risk evaluation.	[27]

## References

[1] Das D., Singha D.L., Paswan R.R., Chowdhury N., Sharma M., Reddy P.S., Chikkaputtaiah C. (2022). Recent advancements in CRISPR/Cas technology for accelerated crop improvement. Planta.

[2] Tang Q., Wang X., Jin X., Peng J., Zhang H., Wang Y. (2023). CRISPR/Cas Technology Revolutionizes Crop Breeding. Plants.

[3] Choudry M.W., Riaz R., Nawaz P., Ashraf M., Ijaz B., Bakhsh A. (2024). CRISPR-Cas9 mediated understanding of plants’ abiotic stress-responsive genes to combat changing climatic patterns. Funct. Integr. Genom..

[4] Cardi T., Murovec J., Bakhsh A., Boniecka J., Bruegmann T., Bull S.E., Eeckhaut T., Fladung M., Galovic V., Linkiewicz A. (2023). CRISPR/Cas-mediated plant genome editing: Outstanding challenges a decade after implementation. Trends Plant Sci..

[5] Sankhla I.S., Kumar A., Singh C.P., Kumar A., Arora S., Ogita S., Yau Y.Y., Mukherjee K. (2024). Nutrient Biofortification in Crop Plants by the CRISPR/Cas9 Technology: A Potential Approach for Sustainable Food Security. Gene Editing in Plants.

[6] Nagamine A., Ezura H. (2022). Genome Editing for Improving Crop Nutrition. Front. Genome Ed..

[7] Kumar R., Das S.P., Choudhury B.U., Kumar A., Prakash N.R., Verma R., Chakraborti M., Devi A.G., Bhattacharjee B., Das R. (2024). Advances in genomic tools for plant breeding: Harnessing DNA molecular markers, genomic selection, and genome editing. Biol. Res..

[8] Abdul Aziz M., Brini F., Rouached H., Masmoudi K. (2022). Genetically engineered crops for sustainably enhanced food production systems. Front. Plant Sci..

[9] Sprink T., Wilhelm R., Ricroch A., Eriksson D., Miladinović D., Sweet J., Van Laere K., Woźniak-Gientka E. (2024). Genome Editing in Biotech Regulations Worldwide. Chapter 25. A Roadmap for Plant Genome Editing.

[10] Turnbull C., Lillemo M., Hvoslef-Eide T.A.K. (2021). Global Regulation of Genetically Modified Crops Amid the Gene Edited Crop Boom—A Review. Front. Plant Sci..

[11] Sánchez M.A. (2020). Chile as a key enabler country for global plant breeding, agricultural innovation, and biotechnology. GM Crops Food.

[12] Text of the Cartagena Protocol on Biosafety. https://bch.cbd.int/protocol/text.

[13] SAG Aplicabilidad de Resolución N° 1.523/2001 en Material de Propagación Desarrollado Por Nuevas Técnicas de Fitomejoramiento. https://www.sag.gob.cl/ambitos-de-accion/aplicabilidad-de-resolucion-ndeg-15232001-en-material-de-propagacion-desarrollado-por-nuevas-tecnicas-de-fitomejoramiento.

[14] Li B., Sun C., Li J., Gao C. (2024). Targeted genome-modification tools and their advanced applications in crop breeding. Nat. Rev. Genet..

[15] Hernández-Soto A., Gatica-Arias A. (2024). Genome editing in Latin America: Research achievements and regulatory evolution. Plant Cell Tissue Organ Cult. (PCTOC).

[16] Sauer N.J., Mozoruk J., Miller R.B., Warburg Z.J., Walker K.A., Beetham P.R., Schöpke C.R., Gocal G.F. (2016). Oligonucleotide-directed mutagenesis for precision gene editing. Plant Biotechnol. J..

[17] Abdelrahman M., Wei Z., Rohila J.S., Zhao K. (2021). Multiplex genome-editing technologies for revolutionizing plant biology and crop improvement. Front. Plant Sci..

[18] Armario Najera V., Twyman R.M., Christou P., Zhu C. (2019). Applications of multiplex genome editing in higher plants. Curr. Opin. Biotechnol..

[19] Hoffman N.E. (2021). Revisions to USDA biotechnology regulations: The SECURE rule. Proc. Natl. Acad. Sci. USA.

[20] CFIA Rationale for Updated Guidelines for Determining Whether a Plant is Regulated Under Part V of the Seeds Regulations (Directive 2009-09). https://inspection.canada.ca/en/plant-varieties/plants-novel-traits/applicants/directive-2009-09/rationale-updated-guidelines.

[21] CTNBIO Tecnologias Inovadoras De Melhoramento Genético (RN16). http://ctnbio.mctic.gov.br/tecnologias-inovadoras-de-melhoramento-genetico-rn16-.

[22] ICA Resolución No. 00022991 (11/11/2022). https://www.ica.gov.co/getattachment/e4b3a97e-b44e-4974-8bb0-f3b947063e67/2022R22991.aspx.

[23] Goberna M.F., Lewi D.M., Godoy P., Simeone F., Esteban Hopp H.E., Abd-Elsalam K.A., Ahmad A. (2024). Regulatory framework for CRISPR- edited crops in Argentina. Chapter 10. Global Regulatory Outlook for CRISPRized Plants.

[24] Biosafety Kenya Genome Editing Guidelines. https://www.biosafetykenya.go.ke/images/GENOME-EDITING-GUIDELINES-FINAL-VERSION-25th-Feb-2022-03.pdf.

[25] Tachikawa M., Matsuo M. (2024). Global regulatory trends of genome editing technology in agriculture and food. Breed. Sci..

[26] Department of Agriculture, The Philippines Memorandum Circular No. 8 2022. https://www.da.gov.ph/wp-content/uploads/2022/06/mc08_s2022_Revised.pdf.

[27] Yang F., Zheng K., Yao Y. (2024). China’s regulatory change toward genome-edited crops. Trends Biotechnol..

[28] USDA/APHIS Confirmation Letters. https://www.aphis.usda.gov/biotech-exemptions/confirmation-letters.

[29] Cibus and Rotam Announce Launch of Their First Non-Transgenic Commercial Product. https://www.cibus.com/press/press111914.php.

[30] ISAAA Gene-Edited High Oleic Soybean Oil Now Available in the US. https://www.isaaa.org/kc/cropbiotechupdate/article/default.asp?ID=17345.

[31] Pairwise Introduces Conscious™ Greens into U.S. Restaurants. https://www.pairwise.com/news/pairwise-introduces-conscious-greens-into-u.s.-restaurants.

[32] GreenVenus™-Romaine Lettuce. https://greenvenusproduce.com/products/lettuce-seeds.

[33] Health Canada List of Non-Novel Products of Plant Breeding for Food Use. https://www.canada.ca/en/health-canada/services/food-nutrition/genetically-modified-foods-other-novel-foods/transparency-initiative/list-non-novel-products-plant-breeding-food-use.html.

[34] OECD Developments in Delegations on the Safety Assessment of Novel Foods and Feeds, May 2023–February 2024. Series on the Safety of Novel Foods and Feeds No. 38..

[35] Kenya News Agency Genome Editing, Game Changer For Healthier and Better Harvests. https://www.kenyanews.go.ke/genome-editing-game-changer-for-healthier-and-better-harvests/.

[36] Heli Z., Hongyu C., Dapeng B., Yee Shin T., Yejun Z., Xi Z., Yingying W. (2022). Recent advances of γ-aminobutyric acid: Physiological and immunity function, enrichment, and metabolic pathway. Front. Nutr..

[37] ISAAA Updates on Regulatory Landscape for Gene-Edited Crops in Asia and Oceania. https://www.isaaa.org/blog/entry/default.asp?BlogDate=8/28/2024.

[38] USDA/FAS MARA Updates Rules for Review of Gene-Edited Plants for Agricultural Use. https://apps.fas.usda.gov/newgainapi/api/Report/DownloadReportByFileName?fileName=MARA%20Updates%20Rules%20for%20Review%20of%20Gene-Edited%20Plants%20for%20Agricultural%20Use%20_Beijing_China%20-%20People%27s%20Republic%20of_CH2023-0080.

[39] 2023 List of Approved Biosafety Certificates for Gene Editing for Agricultural Use (Production Application). http://www.moa.gov.cn/ztzl/zjyqwgz/spxx/202304/P020230428546705804523.pdf.

[40] 2023 Agricultural Gene Editing Biosafety Certificate (Production Application) Approval List. http://www.moa.gov.cn/ztzl/zjyqwgz/spxx/202401/P020240118484537271708.pdf.

[41] 2024 Agricultural Gene Editing Biosafety Certificate (Production Application) Approval List. http://www.moa.gov.cn/ztzl/zjyqwgz/spxx/202405/P020240508640616405262.pdf.

